# Solitary Intestinal Lymphangiectasia Causing Transient Intussusception

**DOI:** 10.7759/cureus.44206

**Published:** 2023-08-27

**Authors:** Luke A Persin, Nadezda Buntic, Gurvinder Kaur, Christopher Yeary, Parisa Vahhabaghai

**Affiliations:** 1 Research, Lincoln Memorial University DeBusk College of Osteopathic Medicine, Harrogate, USA; 2 Internal Medicine, Norton Community Hospital, Norton, USA; 3 General Surgery, Norton Community Hospital, Norton, USA; 4 Pathology and Laboratory Medicine, East Tennessee State University, Johnson City, USA

**Keywords:** colonoscopy, lymphatic malformation, lymphatics, bowel obstruction, lymph, intussusception, lymphangiectasia

## Abstract

Lymphangiectasia is the benign malformation of lymphatic channels associated with either focal or diffuse dilation of vessels and impaired lymph drainage. This malformation has the potential to create a cystic mass due to the accumulation of lymphatic fluid. While rare in adults, intussusception, the telescoping of the proximal bowel into the distal bowel, can be caused by a mass within the bowel. In this case, a near-obstructing cystic colon mass developed in a 74-year-old man; this was later found to be a large lymphangiectasia. In addition, this near-obstructing colonic lymphangiectasia served as the lead point in a colo-colonic intussusception. Due to this complication, the mass was immediately removed by a laparoscopic oncologic right-extended hemicolectomy which proved to be both diagnostic and therapeutic.

## Introduction

Intussusception, the telescoping of the proximal bowel into the distal bowel, is oftentimes considered an urgent surgical issue that can lead to complications such as bowel obstruction and intestinal ischemia. While many cases of intussusception are idiopathic and found in children, masses can serve as lead points of telescoping bowel in adults. This condition in adults can be concerning for potentially malignant processes and must raise concern in clinicians. Of the cases of adult intussusception, 70-90% require definitive treatment, and surgery is most often the treatment of choice [1].

Lymphangiectasia is a benign malformation of lymphatic channels associated with either focal or diffuse dilation of vessels and impaired lymph drainage. In the intestine, these vessels can be located within the mucosa, submucosa, or subserosa [2]. Here, we report a rare case of a large, solitary lymphangiectasia causing transient intussusception with subsequent intermittent colonic obstruction.

## Case presentation

A 74-year-old White male 10 days status post uncomplicated laparoscopic right inguinal hernia repair presented to the emergency department with bleeding from a wound site, right lower quadrant abdominal pain with distension, and testicular pain. He was concerned about surgical site bleeding and scrotal bruising after resuming his warfarin a few days prior. He was experiencing flatus and had a recent non-bloody bowel movement. 

The patient has a history of atrial fibrillation, sick sinus syndrome with a pacemaker, type 2 diabetes mellitus, coronary artery disease, chronic obstructive pulmonary disease, hypertension, and laryngeal cancer treated by radiation (2.5 years prior). He has a history of chronic constipation secondary to opioid use for chronic pain. He reported that he had never had a colonoscopy. 

On physical exam, tenderness and ecchymosis of the right lower quadrant and right side of the scrotum were noted. The surgical incisions were clean, dry, and well-approximated. There was a nontender, nonpulsatile mass upon palpation of the right upper quadrant. All other systems are reviewed and found to be negative.

**Figure 1:**
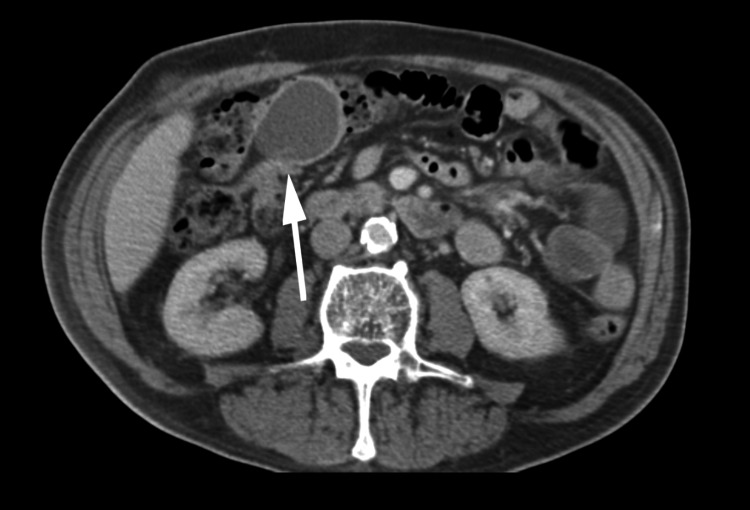
Axial CT showing colocolic intussusception and an ovoid hypodense mass measuring 4.7 x 3.8 cm transaxial by 4.8 cm craniocaudal is seen within the intussusception.

**Figure 2:**
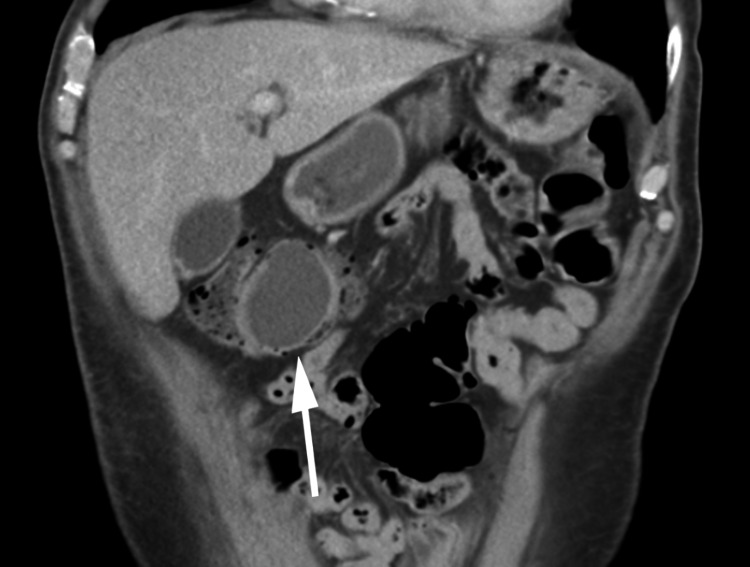
Coronal CT showing colocolic intussusception and an ovoid hypodense mass measuring 4.7 x 3.8 cm transaxial by 4.8 cm craniocaudal is seen within the intussusception.

**Figure 3:**
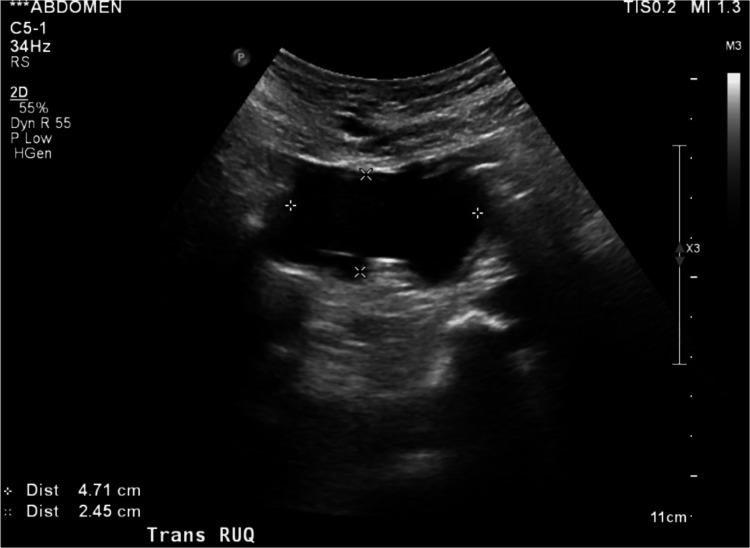
Right upper quadrant abdominal ultrasound showing a 2.5 x 4.7 cm irregular fluid collection near the gallbladder fossa

An abdominal CT with and without contrast from the emergency department showed a colo-colonic intussusception in the hepatic flexure with an ovoid hypodense mass measuring 4.7 x 3.8 cm x 4.8 cm suspicious for being the lead point of the intussusception (Figure 1 and Figure 2). The CT scan showed no signs of ischemia or bowel obstruction. However, a significant stool burden was present proximal to the mass. General Surgery was consulted. Abdominal ultrasound imaging revealed the mass to be a fluid collection near the gallbladder fossa (Figure 3). Improvement of the patient’s pain coupled with a normal bowel movement led us to believe that the intussusception was transient.

**Figure 4:**
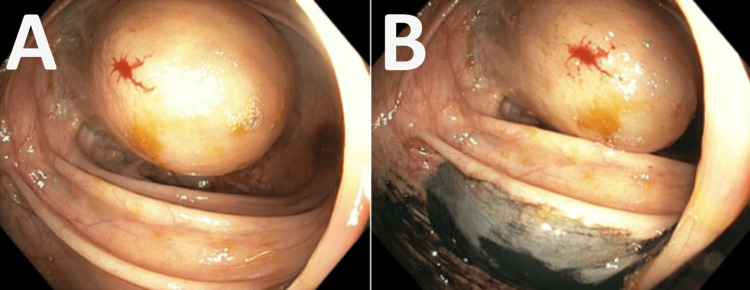
A. Colonoscopic images showing a soft, smooth, polypoid cystic lesion with a broad base. Blood represents the site of biopsy. B. Similar view with dark fluid representing endoscopic tattooing.

A colonoscopy revealed sigmoid diverticulosis and a near-obstructive soft, smooth, polypoid cystic lesion with a broad base located near the hepatic flexure (Figure 4). The lesion was biopsied and tattoo ink was injected at the site. Initial endoscopic pathology results were consistent with colonic mucosa with hyperplastic surface change. Due to the mass nearly obstructing the colon and causing intussusception, a laparoscopic extended right colectomy with ileocolic anastomosis was performed. 

**Figure 5:**
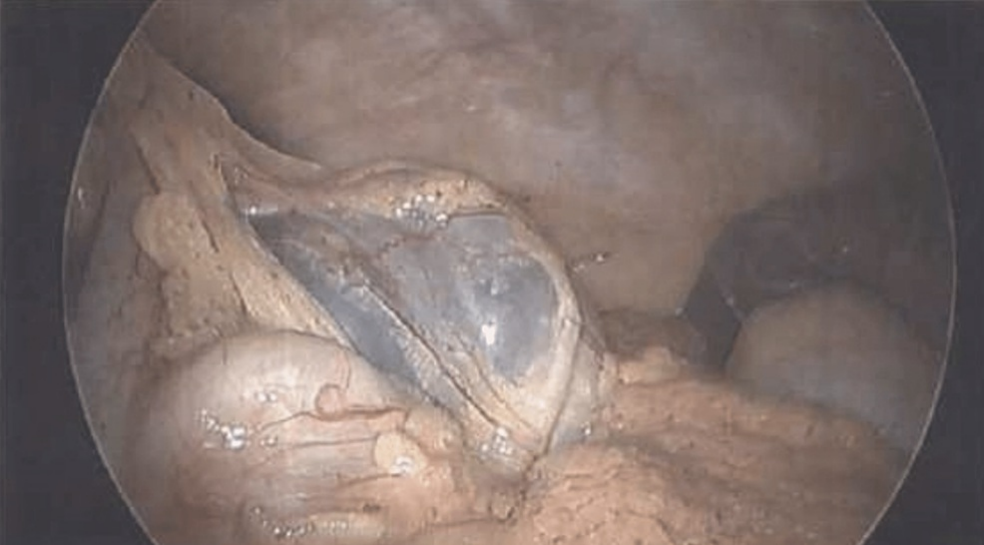
Pathology gross image showing fluid-filled right colon mass during laparoscopic right hemicolectomy

While in the supine position, the patient was put under general anesthesia and intubated. The abdomen was insufflated in normal fashion and we appreciated a right colonic mass with nearby tattooing that did not appear to extend into the circumferential tissue (Figure 5). A laparoscopic oncologic right extended hemicolectomy was performed with 5 cm margins being incorporated into the specimen. Extracorporeal anastomosis was performed with an ileocolonic side-by-side stapled anastomosis. Colonoscopic evaluation was performed and the anastomosis appeared patent and without leaks. Jackson-Pratt drains were placed to bulb suction and the skin was reapproximated using suture. The patient tolerated the procedure well and was discharged home five days postoperative upon resumption of full bowel function and resolution of postoperative pain.

**Figure 6:**
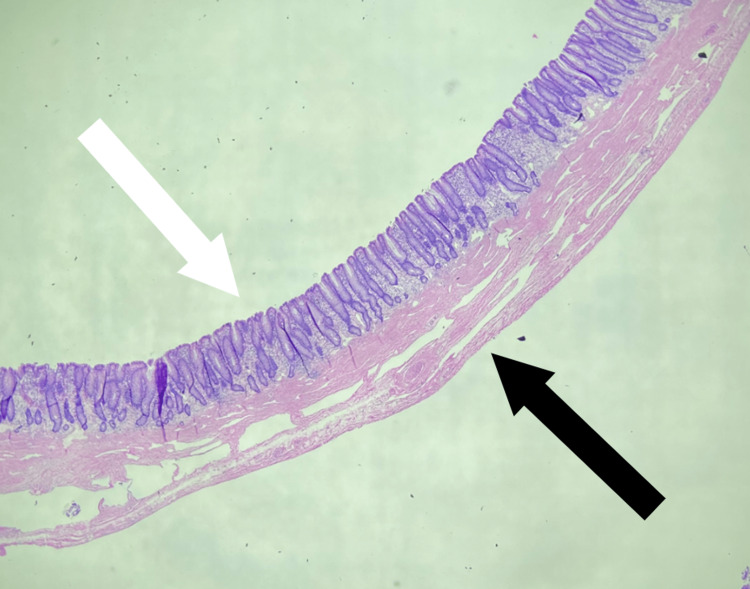
Pathology histological slide showing normal gastrointestinal mucosa (white arrow) and submucosal cyst wall of lymphangiectasia (black arrow)

Final surgical pathology results showed lymphangiectasia forming a thin-walled cystic structure (5.0 cm) with adjacent dilated smaller lymphatic vessels and stromal edema (Figure 6). Additionally, two tubular adenomas and one benign lymph node were appreciated in a distal section of the bowel, unrelated to the lymphangiectasia. No malignancy was identified. The patient followed up in the General Surgery office with a successful recovery back to his baseline health.

## Discussion

The first case of lymphangiectasia was reported by Waldmann et al. in 1961 and since then it has been studied well [3]. The accumulation of lymphatic fluid within the wall of the gastrointestinal tract can arise from two separate mechanisms with either primary or secondary causes [4]. An understanding of each variation of the disease is necessary to understand the full spectrum of its pathology.

The primary type of lymphangiectasia is the result of congenital lymphatic malformation leading to lymphangiomatosis, the presence of diffuse lymphangiectasia in an organ or across multiple organ systems. Patients with this disease have poor lymphatic movement throughout the body. This lymphatic malformation within the intestines is known as “Waldmann’s Disease,” which can cause diffuse small vesicular lymphatic collections on the gastrointestinal mucosa that leak lymphatic fluid into the gastrointestinal tract [5]. This lymphatic drainage can cause a protein-losing enteropathy leading to lymphopenia, hypoalbuminemia, and hypogammaglobulinemia [4]. Patients experience bilateral lower extremity edema, pleural effusions, pericarditis, or chylous ascites and have the potential to develop fatigue, abdominal pain, weight loss, inability to gain weight, diarrhea, and malabsorption [4]. While this presentation of lymphangiectasia has many complications, it fails to produce a large lymphatic obstructing mass within the gastrointestinal system as seen in our patient.

Secondary lymphangiectasia can have a variety of causes; however, it is most often caused by the obstruction of lymphatics. This obstruction impedes the drainage of lymphatic fluid through the thoracic duct into the left subclavian vein. Lymphatic fluid builds up creating a cystic structure that has the potential to burst, creating a protein-losing enteropathy that causes similar complications to those seen in primary lymphangiectasia. Additionally, rupture of such structures has the potential to cause gastrointestinal bleeds [6]. An increase in central venous pressure can impede the drainage of lymphatics thus causing lymphangiectasia. A variety of cardiac causes including right heart failure, constrictive pericarditis, and congenital heart disease have been described in the literature causing lymphangiectasia [7,8]. Hepatic pathologies from portal hypertension or hepatic venous outflow obstructions can also lead to lymphangiectasia through similar obstructing mechanisms. These can be from a variety of etiologies including cirrhosis, transplant, and fibrosis amongst other primary hepatic causes [9]. Malignant pathologies including lymphomas and neuroblastomas have also been reported to cause a secondary lymphatic obstruction leading to lymphangiectasia [10,11]. 

This case is an example of secondary lymphangiectasia. Due to the patient’s age and presentation of a solitary collection of lymphatic fluid, primary lymphangiectasia can be ruled out. While there are many potential causes of secondary lymphangiectasia, there is no clear identifiable source apparent in the case presented. 

Imaging modalities to identify lymphatic malformations have progressed significantly. Traditional CT imaging can show cystic structures but offers little more information regarding lymphatic anatomy due to the limitations of the study. Lymphoscintigraphy with Tc-99m-labeled dextran allows for the accurate tracking of lymphatic collections but fails to present a high-resolution image [12]. Novel magnetic resonance lymphangiography allows for an in-depth evaluation of lymphatic flow disorders with much-improved resolution [13]. In our case, a combination of CT and ultrasound imaging was adequate to identify a large near-obstructing mass but failed to diagnose lymphangiectasia or identify any lymphatic structures. 

There is no true consensus on the management of lymphangiectasia. Treatments vary depending on patient presentation. In focal intestinal lymphangiectasia, surgery or embolization is considered curative. If a patient has diffuse or extensive intestinal lymphangiectasia, surgery is usually not indicated due to the need to resect multiple sections of the bowel [14]. Our patient required urgent surgical consultation due to the resulting intussusception and potential complications.

## Conclusions

Lymphangiectasia, while rare, can present as a near-obstructing colonic mass that can cause other complications such as intussusception. Although not high on the list of differential diagnoses, lymphangiectasia should be suspected when a cystic mass in the abdomen is seen. We report a case of transient colo-colonic intussusception in a 74-year-old man caused by a singular large intestinal lymphangiectasia with a good outcome. Resection of this cystic structure proved both diagnostic and therapeutic, and the patient had no lasting problems. 
